# Trainee Responses to Hurricane Harvey: Correlating Volunteerism With Burnout

**DOI:** 10.3389/fpubh.2018.00224

**Published:** 2018-08-28

**Authors:** Crystal Jing Jing Yeo, Gustavo C. Román, David Kusnerik, Trevor Burt, Dottie Mersinger, Shaylor Thomas, Timothy Boone, Suzanne Z. Powell

**Affiliations:** ^1^Houston Methodist Neurological Institute, Houston, TX, United States; ^2^Graduate Medical Education, Houston Methodist Hospital, Houston, TX, United States

**Keywords:** disaster, trainees, burnout, volunteers, hurricane harvey

## Abstract

**Background:** Natural disasters take a heavy toll not only on their victims, but also on physicians who suffer vicarious trauma and burnout. New trainees in Houston, from entering PGY1 residents to entering fellows, underwent even more upheaval and stress during Hurricane Harvey. Many responded to calls for volunteer help.

**Objective:** To investigate the impact of Hurricane Harvey on new trainees at our institution, and correlate volunteerism with measures of burnout and resilience.

**Methodology:** Thirty three new trainees out of 90 (43% of population) from all specialties in our institution voluntarily responded to an online survey on the impact of Hurricane Harvey on their lives, whether or not they volunteered and in what form, and answered questions drawing from the abbreviated Maslach burnout survey and Resiliency Quiz. Statistical analyses were conducted using GraphPad Prism and Excel data analysis.

**Results:** The top areas impacted were emotional health (32%), eating habits (29%), family (25%) and finances (25%). The main voluntary activities were covering for colleagues who could not make it to hospital (50%), donating money and supplies (36%), and cleaning and rebuilding (36%). Volunteering was associated with feelings of appreciation (76%), happiness (62%), thankfulness (57%), purposefulness (43%) and pride (33%). Fewer volunteers scored lowly in personal achievement as compared to non-volunteers (10 vs. 38%, *p* = 0.05).

**Significance:** Hurricane Harvey affected health, finances and family of new trainees, more than half of whom volunteered to help. Volunteers had a greater sense of personal achievement as compared to non-volunteers. This may be due to having more volunteers among less burnt-out trainees or because volunteering reduced burnout and stress responses/trauma. These results suggest that volunteer opportunities should be made available in programs targeting resident burnout.

## Introduction

**Figure 1:**
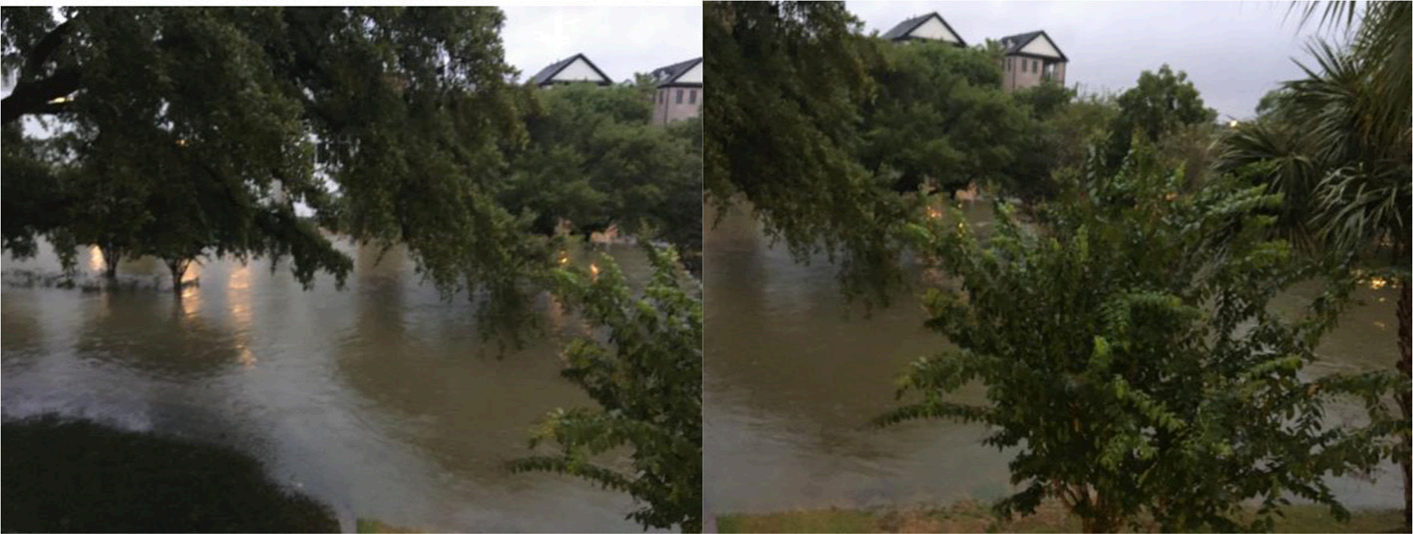
Roads completely submerged by floodwaters near the Texas Medical Center (Crystal Yeo).

In August 2017, the tropical cyclone, Hurricane Harvey, caused widespread flooding and massive destruction to the Houston metropolitan area. The Texas Medical Center, the largest medical center in the world and home to numerous residency and fellowship programs, was flooded in (Figure 1). Healthcare providers who had taken responsibilities as members of the “ride-out” teams were unable to leave campus for days because of surrounding floods. Many had to provide coverage until relief staff could reach the hospitals. Patients were unable to receive adequate home or medical care, access regular dialysis or chemotherapy treatments, refill their medications, and suffered post-traumatic stress. For weeks after the flooding subsided, patients visited the hospitals for emergency treatment precipitated by lack of self-care or medical treatment during the natural disaster, or could not leave the hospital as they had nowhere to go.

Natural disasters take a heavy toll not only on their victims, but also on the physicians who experience the impact through their patients and suffer vicarious traumatization and burnout (1). Vicarious traumatization refers to the emotional and psychological reactions triggered by the experience of empathic engagement with patients who are survivors of trauma. Most healthcare providers were affected by Hurricane Harvey. Many had to evacuate their homes, or knew relatives and loved ones who had to do so and had lost their livelihoods, or had experienced the loss of friends and family. Although long work hours and stressful work conditions are expected in training in many medical specialties, the disaster left healthcare workers working in unprecedented and extreme circumstances. The emotional toll on physicians and trainees continuing to treat a traumatized population in this situation was immense. In addition, new trainees experienced an even more significant emotional toll as they had started their residencies and fellowships less than 2 months prior to the disaster, and were already dealing with adjustments related to relocation and new professional responsibilities. Such psychological stressors put the trainees at greater risk of maladaptive coping responses, neglect of self-care, and burnout.

Physician burnout is a growing epidemic which has been reported in every specialty and every level of training from medical students to residents to attending physicians (2). Burnout is a multi-dimensional syndrome defined as “emotional exhaustion, depersonalization and reduced personal accomplishment” typically associated with workplace stress (3). It is caused by one or more of the following: high levels of emotional exhaustion, a chronic state of physical and emotional fatigue resulting from excessive professional and personal demands, high levels of depersonalization, a set of callous and insensitive behaviors toward patients, coworkers and oneself, and low levels of personal achievement, a set of feelings of incompetence and low self-esteem. Physicians who are burnt-out are unlikely to engage in quality patient care or contribute to a positive learning environment, leading to poorer professionalism, worse workplace performance, increased medical errors, and poorer patient outcomes. Training programs which prevent resident burnout and integrate wellness may enhance trainees' learning and ability to fulfill the 6 core competencies, as described by ACGME. These programs may focus on implementing measures which prevent and/or address burn-out, such as ways to increase resilience, which is defined as the capacity to respond to stress in a healthy way such that goals are achieved at minimal psychological and physical costs (4). Examples of resident wellness programs include weekly arts classes at the Mayo clinic, yoga classes, protected therapist time, mentoring and bonding activities at Stanford, and community service and bonding activities at Vanderbilt and the University of Alabama. These programs are mirrored at our institution, which provides stress management, healthy eating and educational programs to all employees, including trainees and physicians. These include massage therapies, stress consultations and management classes which teach breathing, visualization, affirmations and mindfulness, yoga classes, aerobics classes, acupuncture, sportsmetrics, lifestyle coaches and nutrition consults. Volunteering has been associated with improved mental and physical health, an “escape hatch” from regular constrains of physician work, and may serve as an inoculation against trainee and physician burnout (5). We sought to investigate the impact of Hurricane Harvey on new trainees at our institute, and correlate volunteerism with measures of burnout and resilience.

## Methods

This cross-sectional survey was carried out 2 weeks after Hurricane Harvey on 33 first-year trainees at a single academic hospital at the Texas Medical Center. All first-year trainees were eligible to participate in this survey. Institutional IRB approval was obtained for the study. Trainees were informed that they could refuse to participate and specifically told that non-participation would not affect their evaluation by program directors and faculty. In addition, they were informed that each resident would be assigned a code, all information would be de-identified, privacy and confidentially would be maintained and at no point would this information be made known to their faculty or evaluators. If they wished to seek medical or psychological help, information would be provided to them by study investigators on where they could seek confidential help, such as the ombudsmen, counseling services or external psychologists. Thirty three new trainees out of ninety (43%), from all specialties in our institution, voluntarily responded to take an online survey on the impact of Hurricane Harvey on their lives, whether they volunteered and in what form, and answered questions assessing burnout and resilience. To evaluate the generalizability of this population to the overall first-year population, their demographics and survey responses were compared to that of 47 new trainees out of 90 (52%) who had volunteered only to take an online survey on questions assessing burnout and resilience. To evaluate measures which correlated with psychological distress, another cohort of 27 first-year trainees volunteered to answer survey questions on psychological distress, burnout and resilience.

### Measures

Demographics collected included age, sex, highest academic degree, specialty, marital status, and the presence of children in the family. The abbreviated, 9-item form of the Maslach Burnout Inventory (MBI), which has previously been used for burnout evaluation in physicians, was used to measure burnout (3). Response options used a 7-point Likert scale ranging from 0 to 6 Three subscales of burnout (emotional exhaustion, depersonalization, and personal accomplishment) were evaluated with this inventory and scored separately. The Resiliency Quiz, available at http://resiliencyquiz.com/index.shtml, a 20-item questionnaire developed by the Resiliency Center, was used to evaluate resilience using a 5-point Likert scale ranging from 1 to 5. The Kessler Psychological Distress Scale (K10), a 10 item questionnaire intended to yield a global measure of distress based on questions about anxiety and depressive symptoms that a person has experienced in the most recent 4-week period, was used to measure anxiety and depression, using a 5-point Likert scale ranging from 1 to 5. High scorers on each of the parameters of the Resiliency Quiz and abbreviated MBI were defined as more than 75 percentile (6), low scorers were defined as less than 25 percentile. High scorers on the K10 were defined as more than 20. An abbreviated consent was incorporated to the first page of the online survey.

### Data collection and analysis

Survey Monkey was used to administer online surveys and collect results for rapid analysis. Univariate analysis performed using Excel data analysis showed that skewness and kurtosis values of the populations were between −2 to +2, within limits of normal distributions. Means were compared using unpaired *t*-test and proportions were compared using N-1 Chi-squared test. Linear regression was used to compare the associations between different scores. Statistical analyses were conducted using Excel and GraphPad Prism.

## Results

Thirty-three new trainees enrolled in this cross-sectional study. The mean age was 30, with slightly more male distribution at 61%. About a third were single, another third married with children, and another third married without children. Half were in medicine specialties, including internal medicine, family medicine and neurology, one third were in surgical specialties including general surgery, orthopedics, obstetrics and gynecology, urology and neurosurgery, and the remainder were in pathology. These demographics and specialty distributions were similar across the study cohort and its volunteers and non-volunteers, as well as the 47 survey-takers from the 90 strong first-year cohort (Table 1).

**Table 1:** Demographics and specialty distribution among volunteers, non-volunteers, the study cohort, and responders from the first year cohort were similar and had no significant differences.

**Cohorts**	**Age**	**Male**	**Female**	**Single**	**Married without children**	**Married with children**	**Medicine**	**Surgery**	**Pathology**
Volunteer	29.8	0.65	0.35	0.25	0.35	0.40	0.45	0.40	0.15
Non volunteer	29.8	0.54	0.46	0.23	0.46	0.23	0.54	0.23	0.23
Study cohort	29.8	0.61	0.39	0.27	0.39	0.33	0.48	0.33	0.18
First year cohort	29.7	0.60	0.40	0.32	0.38	0.28	0.53	0.32	0.15

**Figure 2:**
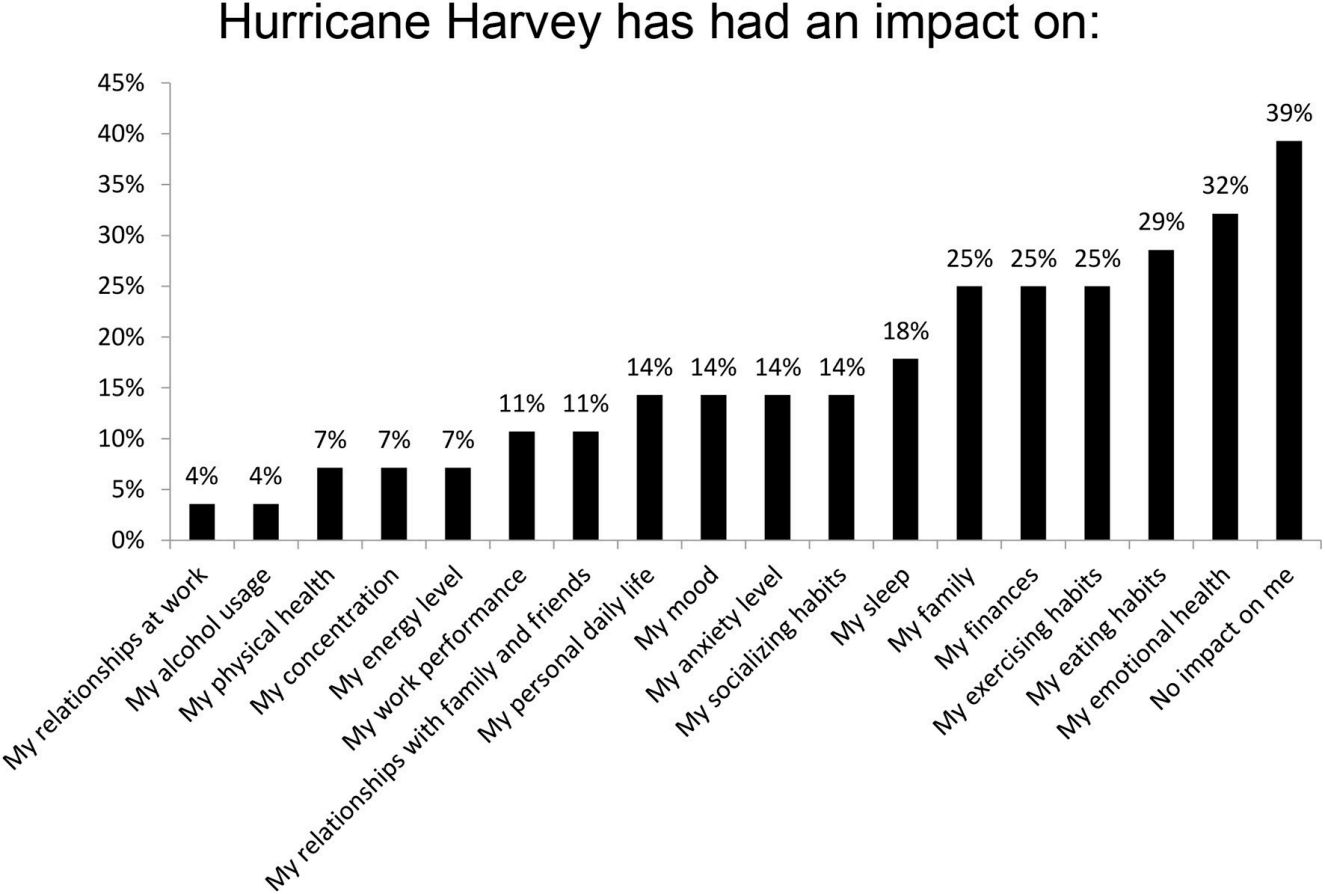
Impact of Hurricane Harvey on new trainees. Survey responders were able to choose any and all of the options which applied to them.

**Figure 3:**
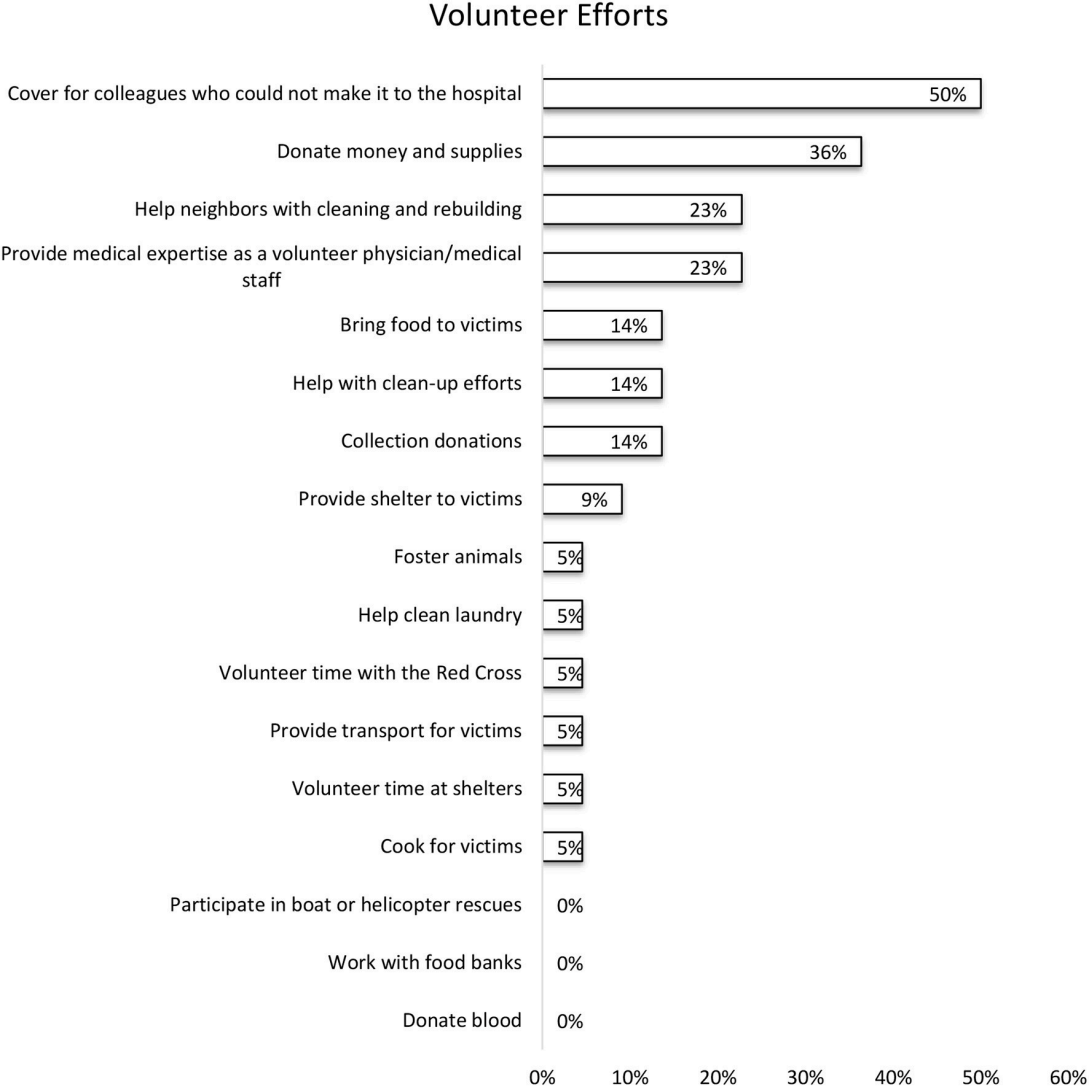
Types of volunteer activities the trainees participated in. The survey responders were able to choose any and all activities which they participated in.

**Figure 4:**
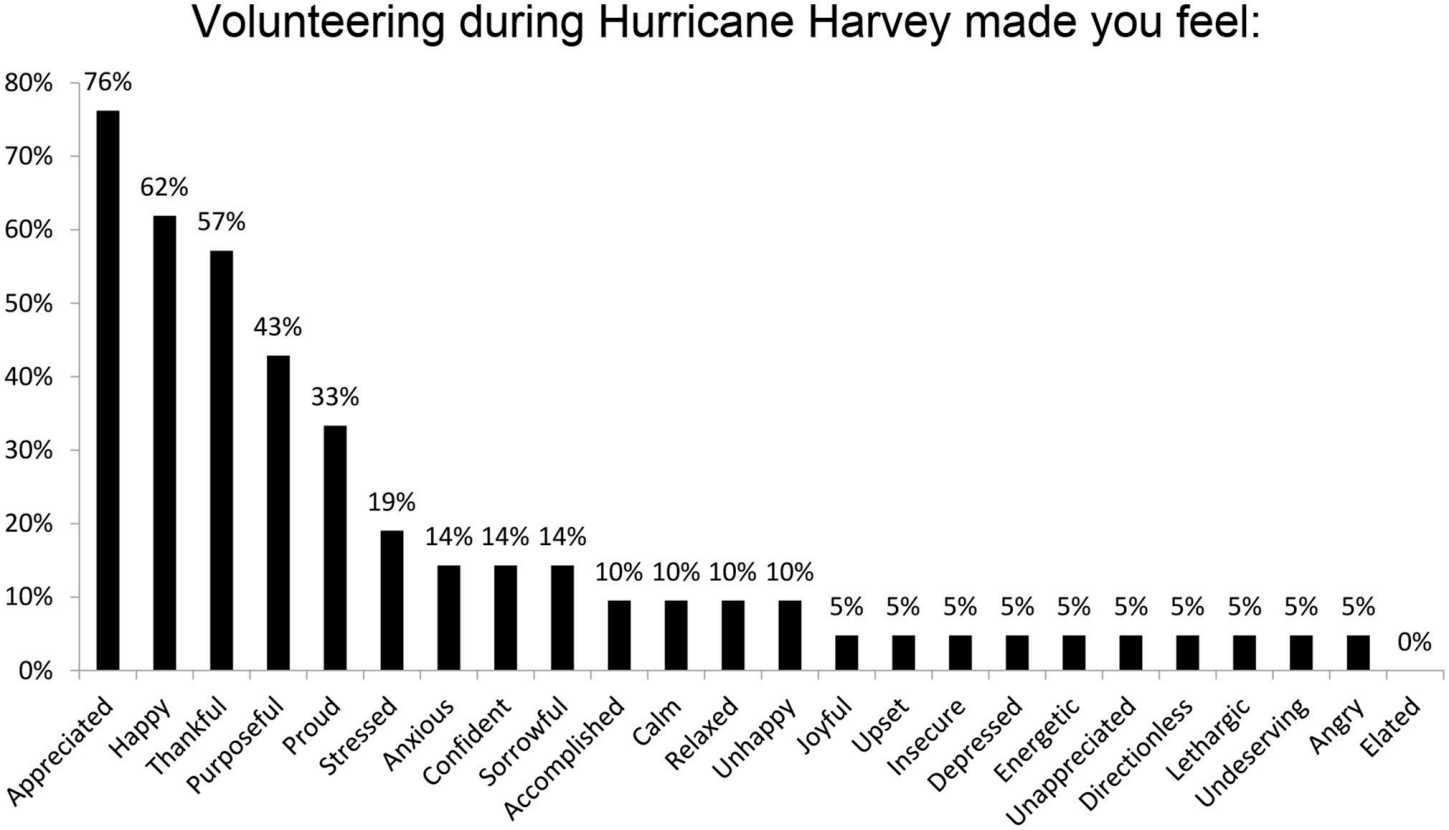
Volunteerism led to greater feelings of appreciation and well-being. Survey responders were able to select any and all of the feelings which applied.

Sixty-one percent (20 of the 23) new trainees were affected by Hurricane Harvey. The aspects of life which were most impacted by Hurricane Harvey were the trainees' emotional health (32%), eating habits (29%), family (25%), finances (25%), and sleep (18%). Changes in alcohol usage (3.57%) and work relationships (3.57%) were the least affected (Figure 2). Of the responders, 20 had volunteered help and 13 did not. As depicted in Figure 3, the top voluntary activities were covering for colleagues who could not make it to hospital (50%), donating money and supplies (36%), helping neighbors with cleaning and rebuilding (23%) and volunteering as a physician or medical staff (23%). Volunteering was associated with feelings of being appreciated (76%), happiness (62%), thankfulness (57%), purposefulness (43%), and pride (33%) (Figure 4).

The means of scores in personal achievement, emotional exhaustion, depersonalization and resilience in the study cohort were 14.2, 8.1, 4.5, and 69.1, which were similar to the first-year cohort (Table 2). The distribution of high and low scorers was also similar between study and first-year cohort. In the study cohort, 21% were low scorers in personal achievement, 9% were high scorers in emotional exhaustion, 15% were high scorers in depersonalization, 12% were low scorers in resilience, and 24% were high scorers in resilience (Table 3).

**Table 2:** Comparison of means of burnout and resilience measures across survey takers from the first-year cohort, current study cohort, volunteers and non-volunteers from the current study cohort showed no significant difference.

**Survey Takers**	**Personal Achievement**	**Emotional Exhaustion**	**Depersonalization**	**Resilience**
First-year cohort, *n* = 47	14.6	10.2	4.3	73.1
Study cohort, *n* = 33	14.2	8.1	4.5	69.1
Volunteers	15.1	10.4	4.5	72.9
Non volunteers	13.7	9.8	4.0	73.8

**Table 3:** Comparison of high and low scorers on burnout and resilience measures across survey takers from the first-year cohort and current study cohort show no significant difference.

**Proportion**	**First year cohort, *n* = 47**	**Study cohort, *n* = 33**	***P***
High resilience	0.17	0.24	0.44
Low resilience	0.19	0.12	0.40
High personal achievement	0.23	0.12	0.22
Low personal achievement	0.26	0.21	0.60
High emotional exhaustion	0.19	0.09	0.22
Low emotional exhaustion	0.23	0.21	0.83
High depersonalization	0.15	0.15	1.00
Low depersonalization	0.13	0.21	0.34

The percentage of low scorers in personal achievement was significantly lower in the volunteers as compared to non-volunteers (10 vs. 38%, *p* = 0.05) (Table 4). The percentage of high scorers in personal achievement was higher in the volunteers as compared to non-volunteers (20% vs. 0%, p=0.09), however the finding did not meet statistical significance. The high and low scorers in emotional exhaustion, depersonalization and resilience did not differ significantly.

**Table 4:** Comparison of high and low scorers on burnout and resilience measures across volunteers and low volunteers show a significantly lower proportion of low scorers in personal achievement in the volunteers as compared to non-volunteers, *P* = 0.05.

**Measures**	**Volunteers**	**Non-volunteers**	***P***
High personal achievement	0.20	0.00	0.09
Low personal achievement	0.10	0.38	^*^0.05
High emotional exhaustion	0.05	0.15	0.33
Low emotional exhaustion	0.15	0.31	0.31
High depersonalization	0.20	0.08	0.22
Low depersonalization	0.25	0.15	0.49
High resilience	0.25	0.23	N.A.
Low resilience	0.15	0.08	0.53

* *Statistically significant*.

**Figure 5:**
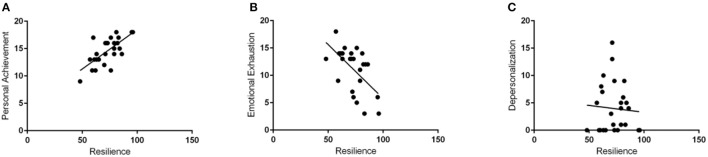
Resilience positively correlates with personal achievement and negatively with emotional exhaustion. Resilience scores on the Resiliency Quiz correlated positively with Personal Achievement scores (**A**, *p* < 0.001) and negatively with emotional exhaustion scores (**B**, *p* = 0.015). The correlation with depersonalization was non-significant **(C)**.

**Figure 6:**
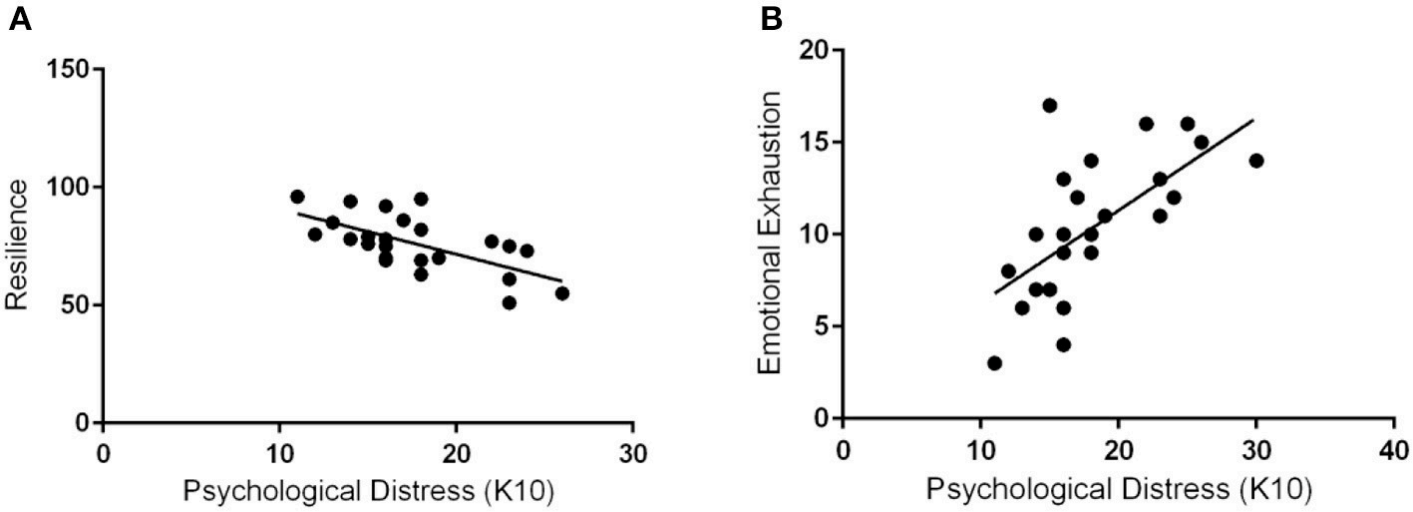
Psychological distress negatively correlates with resilience and positively correlates with emotional exhaustion. Scores on the Kessler Psychological Distress Scale (K10) correlated negatively with scores on the Resiliency Quiz (**A**, *p* < 0.001) and positively with emotional exhaustion scores (**B**, *p* < 0.001).

Personal achievement correlated significantly with resilience (*p* < 0.001) (Figure 5). Resilience also significantly correlated negatively with emotional exhaustion (*p* = 0.0015). There was no correlation with depersonalization. Psychological distress correlated significantly in a positive trend with emotional distress and in a negative trend with resilience (*p* < 0.001) (Figure 6).

## Discussion

Hurricane Harvey had a significant self-reported emotional impact on the new trainees, who are at risk of suffering burnout through vicarious traumatization from the natural disaster, in addition to the intrinsic stressors of starting residency, such as the fears of making mistakes with serious consequences, work overload, and working with uncooperative colleagues (7). Together with many of the Houston population, more than half of the new trainees surveyed had volunteered to provide both medical and non-medical help for victims of the hurricane and its resultant flood. Volunteers reported feelings of appreciation, well-being and purpose, and a greater sense of personal achievement.

Maslach and colleagues defined “burnout” by 3 parameters: emotional exhaustion, in which overwhelming stress and demands deplete one's energy; depersonalization, in which one becomes cynical and detached; and feelings of incompetence, in which one perceives a lack of personal achievement (3). While some studies have focused on emotional exhaustion with or without depersonalization and personal achievement to define burnout (6), since emotional exhaustion is strongly associated with depression (8), all 3 parameters may exist separately or together on a spectrum (3). Our finding that psychological distress positively correlates with emotional exhaustion is consistent with studies correlating emotional exhaustion with stress and depression (8). We have also shown that more non-volunteers report feelings of lack of personal achievement as compared to volunteers.

Given that physician burnout adversely affects quality of patient care and drives attrition from the workforce, addressing lack of personal achievement is important, since these feelings have been linked to medical errors and poor-quality patient care in attending physicians (9), as well as decreased job satisfaction in academic faculty (10). Residents with burnout perceive that they are less competent, commit more medical errors and are poorer at patient care (11). Conversely, those who perceive greater personal achievement are less burnt-out. Factors which increase personal achievement include constructive feedback, good social support, and feeling valued, implying that workplace and training adjustments can change feelings of personal achievement (12)_._ Since we have shown that personal achievement strongly correlates with resilience, and resilience negatively correlates with psychological distress, implementing measures to increase feelings of competency and personal achievement may be one way to increase resilience and decrease burnout and depression among residents.

A critical natural disaster and its ramifications leads to a situation of immense stress, amplified in healthcare professionals by vicarious traumatization. Overwhelming stress, in the absence of coping skills, can lead to distress and burnout (13). Personal attributes such as emotional intelligence, grit and resilience may help one to cope with overwhelming stress, mitigate its effects, and protect against burnout (14, 15). While resilience is thought to be a phenotype derived from complex life experiences, it is possible to design stress-coping interventions to increase resilience (16, 17).

Volunteering has been shown to improve physical and mental well-being and reduce mortality (18, 19). This has been associated with the personal sense of accomplishment and purpose which one gains from volunteer activities (20, 21). Physicians generally have positive experiences with volunteering at community or safety net clinics, and the ability to focus more on patients, avoid organizational problems such as electronic medical records and regulations, and have more control over hours. These have been shown to improve well-being and protect against feelings of burnout (5, 22). The main difficulty for physicians who wish to volunteer is the lack of time. Graduate Medical Education (GME) offices and residency programs can address this by setting aside protected time for trainee volunteer programs throughout residency training. In addition, GME and residency programs can consider adding structured and safe volunteering as part of emergent disaster management plans, and provide opportunities for short-term global medicine volunteering to prevent burnout and vicarious traumatization.

The limitations of this study are mainly due to design. As this is a cross-sectional survey, we can only hypothesize causality from the associations found. Our response rate is approximately 43%, based on the total number of new trainees entering our institution. Selection bias should be kept in mind when interpreting the results. However, the demographics, burnout and resiliency scores of the study cohort and a larger first-year survey cohort are similar. While making the survey mandatory or linking it to the ACGME program evaluation may have provided greater response rates, the privacy and voluntary participation of the trainees were given the highest priority in terms of response.

Future directions would include enrolling a bigger sample size to provide more statistical power, by including trainees at all levels, physicians, and other medical staff. Given that this is already the fourth such natural disaster impacting Houston in almost 20 years, with Tropical Storm Allison in 2001, Hurricanes Katrina and Rita in 2005 and Hurricane Irma in 2008, it would be highly relevant for the study cohort to be extended to all physicians and trainees in the Texas Medical Center. This would enable all institutions in the area to collaborate on lessons learned and optimize critical disaster management plans. Our findings suggest that there are benefits of including volunteer work in disaster management plans. A prospective randomized control trial would be instrumental in evaluating the relationship between volunteering, personal achievement, resilience and psychological distress.

## Conclusion

Natural disasters are highly stressful events which take a heavy toll not only on their victims, but also on the physicians who experience the impact through their patients and suffer vicarious traumatization and burnout. The once in a life-time impact of Hurricane Harvey on new trainees was explored in this cross-sectional survey. Results showed a significant association between volunteerism and a greater sense of personal achievement. Personal achievement was strongly associated with resilience, which in turn was protective against psychological distress. While it is not possible to infer causality in this study; that is, whether the associations were because more resilient and less burnt-out trainees volunteered, or because volunteering increased resilience and reduced burnout, this study suggests that structured volunteer opportunities during critical disaster management and beyond should be explored. These may be offered not just in Graduate Medical Education programs targeting resident burnout, but also in wellness programs for attending physicians and other medical staff.

## Ethics statement

This study was carried out in accordance with the recommendations of IRB, Houston Methodist Hospital. The protocol was approved by the IRB committee. All subjects gave informed consent in accordance with the Declaration of Helsinki.

## Author contributions

CY and SP conceptualized and implemented the project, and authored the paper. GR contributed to the conceptualization, project implementation, and writing of the paper. DK, TB, DM, ST, and TB contributed to the implementation of the project.

### Conflict of interest statement

The authors declare that the research was conducted in the absence of any commercial or financial relationships that could be construed as a potential conflict of interest.
